# Aerosol Delivery of Surfactant Liposomes for Management of Pulmonary Fibrosis: An Approach Supporting Pulmonary Mechanics

**DOI:** 10.3390/pharmaceutics13111851

**Published:** 2021-11-03

**Authors:** Sabna Kotta, Hibah Mubarak Aldawsari, Shaimaa M. Badr-Eldin, Lenah S. Binmahfouz, Rana Bakur Bakhaidar, Nagaraja Sreeharsha, Anroop B. Nair, Chandramouli Ramnarayanan

**Affiliations:** 1 Department of Pharmaceutics, Faculty of Pharmacy, King Abdulaziz University, Jeddah 21589, Saudi Arabia; haldosari@kau.edu.sa (H.M.A.); smbali@kau.edu.sa (S.M.B.-E.); rbakhaidar@kau.edu.sa (R.B.B.); 2 Center of Excellence for Drug Research and Pharmaceutical Industries, King Abdulaziz University, Jeddah 21589, Saudi Arabia; 3 Department of Pharmacology and Toxicology, Faculty of Pharmacy, King Abdulaziz University, Jeddah 21589, Saudi Arabia; lbinmahfouz@kau.edu.sa; 4 Department of Pharmaceutical Sciences, College of Clinical Pharmacy, King Faisal University, Al-Ahsa 31982, Saudi Arabia; sharsha@kfu.edu.sa (N.S.); anair@kfu.edu.sa (A.B.N.); 5 Department of Pharmaceutics, Vidya Siri College of Pharmacy, Off Sarjapura Road, Bangalore 560035, India; 6 Department of Pharmaceutical Chemistry, Vidya Siri College of Pharmacy, Off Sarjapura Road, Bangalore 560035, India; pharmwhiz@gmail.com; 7 Global Technical Enablement JMP Division, SAS India Pvt. Ltd., Lavelle Road, Bengaluru 560025, India

**Keywords:** pulmonary fibrosis, naringin, bleomycin, liposomes, aerosol

## Abstract

Excessive architectural re-modeling of tissues in pulmonary fibrosis due to proliferation of myofibroblasts and deposition of extracellular matrix adversely affects the elasticity of the alveoli and lung function. Progressively destructive chronic inflammatory disease, therefore, necessitates safe and effective non-invasive airway delivery that can reach deep alveoli, restore the surfactant function and reduce oxidative stress. We designed an endogenous surfactant-based liposomal delivery system of naringin to be delivered as an aerosol that supports pulmonary mechanics for the management of pulmonary fibrosis. Phosphatidylcholine-based liposomes showed 91.5 ± 2.4% encapsulation of naringin, with a mean size of 171.4 ± 5.8 nm and zeta potential of −15.5 ± 1.3 mV. Liposomes with the unilamellar structure were found to be spherical and homogeneous in shape using electron microscope imaging. The formulation showed surface tension of 32.6 ± 0.96 mN/m and was able to maintain airway patency of 97 ± 2.5% for a 120 s test period ensuring the effective opening of lung capillaries and deep lung delivery. In vitro lung deposition utilizing Twin Stage Impinger showed 79 ± 1.5% deposition in lower airways, and Anderson Cascade Impactor deposition revealed a mass median aerodynamic diameter of 2.35 ± 1.02 μm for the aerosolized formulation. In vivo efficacy of the developed formulation was analyzed in bleomycin-induced lung fibrosis model in rats after administration by the inhalation route. Lactate dehydrogenase activity, total protein content, and inflammatory cell infiltration in broncho-alveolar lavage fluid were substantially reduced by liposomal naringin. Oxidative stress was minimized as observed from levels of antioxidant enzymes. Masson’s Trichrome staining of lung tissue revealed significant amelioration of histological changes and lesser deposition of collagen. Overall results indicated the therapeutic potential of the developed non-invasive aerosol formulation for the effective management of pulmonary fibrosis.

## 1. Introduction

Airway delivery of therapeutics as nano-formulation has been demonstrated to be a viable approach for site-specific of medicines for treating chronic, progressive, and fatal lung diseases like fibrosis [1]. In pulmonary fibrosis, the proliferation of myofibroblasts and deposition of extracellular matrix cause reconstruction of lung tissue affecting elasticity of the alveoli that reduces lung function [2]. Being a chronic disease, pulmonary fibrosis needs long-term treatment and rationalizes the quest for the local delivery of novel and safe anti-inflammatory and anti-fibrotic medications. Local delivery of nanotherapeutics improves biodistribution and reduces systemic toxicity as compared to conventional formulations administered intravenously [3].

The focus of the research has always been the use of nano-carriers for aerosol delivery that will eventually lead to the improved therapeutic efficacy of the drug [4]. However, being a treatment of vital organs, the material-borne toxicity of such carriers should not be overlooked [5]. This concern becomes more important as many nanoparticulate systems have shown pulmonary toxicity after inhalation. Effective clinical translation of therapeutically effective aerosol drug delivery would thus need a biocompatible and safe nano-carrier.

Pulmonary surfactant is the most important biological barrier of the respiratory surface, as it restricts the entry of foreign particles into the lungs. Native pulmonary surfactant consists of a few surfactant proteins and various lipids. Phosphatidylcholine constitutes the major fraction of the lung surfactant [6]. We hypothesized that liposomes engineered using safe, biocompatible endogenous pulmonary surfactant mimetic lipids could support pulmonary mechanics by maintaining airway patency and achieving deep alveolar reach without exhibiting pulmonary toxicity will be a possible approach for efficient yet safe aerosol delivery of the anti-fibrotic drug.

Recently approved nintedanib and pirfenidone are anti-fibrotic drugs that slow down the loss of lung function; however, they do not have a significant effect in alleviating patient symptoms [7]. Long-term oral therapy results in several severe side effects, predominantly gastrointestinal disturbances [8]. Neither of these medications helps reverse lung fibrosis because the exact etiology of pulmonary fibrosis is complex and still not understood completely [9]. Fibrotic changes occur as a result of oxidative stress and the release of a plethora of cytokines, enzymes like proteases, peroxidases, and several growth factors [10]. Naringin, an active polyphenolic bioflavonoid from various fruits of the citrus family, has demonstrated potential anti-inflammatory effects in vitro as well as in vivo and also reduces oxidative stress-related diseases [10,11]. Considering the beneficial effect of naringin on pulmonary fibrosis, we designed a convenient aerosol system that is pulmonary mechanics compatible and can efficiently reach deep alveoli for a better therapeutic effect.

The present mechanistic exploration study provides a validated ‘proof-of-concept’ for the efficient localized therapy of pulmonary fibrosis using bioactive phytoconstituent naringin delivered by aerosol. Being amphipathic, endogenous surface-active phospholipid-based liposomal nano-carriers of naringin will decrease the surface tension at the alveolar surface and therefore keep airway patency intact. Thus, for the treatment of pulmonary fibrosis, a large payload of lipophilic naringin may be given to the lungs through aerosol administration. We characterized liposomal nano-carriers for aerosol-based administration for various physicochemical parameters like particle size, zeta (ζ) potential, and imaging through an electron microscope. To validate the appropriateness for aerosol delivery for inhalation, we assessed the airway patency, *in* mass median aerodynamic diameter (MMAD), and in vitro lung deposition pattern. Further, in vivo effectiveness tests in bleomycin-induced animal models strengthened the applicability of aerosol-based delivery systems as a viable approach for the treatment of pulmonary fibrosis.

## 2. Materials and Methods

### 2.1. Materials

Lipoid GmbH, Ludwigshafen, Germany, provided Phospholipon 90 G (95.6 percent Phosphatidylcholine) as a gift sample, which was utilized without additional purification. Liposomes were prepared using HPLC-grade chloroform and methanol. Naringin, D-α-tocopheryl polyethylene glycol 1000 succinate (TPGS), Acetonitrile (HPLC), Cholesterol, Bleomycin, Formaldehyde, Orthophosphoric acid, Potassium chloride, Calcium chloride, Sodium chloride, hematoxylin-eosin (H and E) stain and Biochemistry analysis kits, Masson’s Trichrome stain were purchased from Sigma Aldrich (Bangalore, Karnataka, India) and Cayman Chemical Company (Ann Arbor, MI, USA).

### 2.2. Methods

#### 2.2.1. Formulation of Naringin Liposomes

Liposomes were produced via the thin-film hydration technique [12]. Briefly, Phospholipon 90 G, cholesterol and naringin (9:1:1) were dissolved in methanol:chloroform (1:2 *v*/*v*) mixture and dried at 40 °C in a rotary vacuum evaporator (Rotavapor R-300, BUCHI India Pvt Ltd. Mumbai, Maharashtra, India) to form a thin film [13]. The film was kept overnight under vacuum to remove traces of organic solvent and then hydrated with 0.9% saline (pH 7.4) containing TPGS (0.25% *w*/*v*) at 40 °C for 60 min with continuous stirring yielding liposomal suspension of concentration 40 mg lipid/mL suspension. Samples were passed through a high-pressure homogenizer (Panda 2K, GEA Niro Soavi, Parma, Italy) fitted with a shell and tube heat exchanger. The homogenization was optimized and programmed to 5 cycles at 100 bars followed by 5 cycles at 200 bars to get small unilamellar liposomes.

#### 2.2.2. Physicochemical Characterization of Naringin Liposomes—Electron Microscopy, Zeta Potential, Particle Size, PDI, and Encapsulation Efficiency

For physical characterization, the liposome sample was diluted with hydration media to produce 1 mg phospholipid/mL and added to a clear polystyrene cuvette (path length = 1 cm). The cuvette was placed in a thermostatic sample chamber. The mean particle size and polydispersity index of the resulting liposome dispersion were measured using photon cross-correlation spectroscopy (Nanophox, Sympatec, Clausthal-Zellerfeld, Lower Saxony, Germany), with detection at a scattering angle of 90°. Three runs of 60 s were performed at 25 °C, and the mean particle size was calculated [14]. Zeta potential was evaluated using a zeta potential analyzer (DelsaNano C, Beckman Coulter, Tokyo, Japan) (DelsaNano C, Beckman Coulter, Tokyo, Japan) [15].

Scanning electron microscopy (SEM) was performed by placing the liposomal sample on carbon conductive adhesive tape. The tape was placed on the specimen stub. The sample was seen at an accelerating voltage of 5.0 kV in the microscope (JSM-7600F Field Emission Gun SEM) fitted with a cryo unit (PP3000T) (Quorum Technologies, Laughton, East Sussex, UK) (Quorum Technologies, Laughton, UK) [16].

For capturing pictures using transmission electron microscopy (TEM, JEM 2100, JEOL, Tokyo, Japan), the liposomal sample was placed on a carbon-coated formvar grid. Previously neutralized Phosphotungstic acid (2% *w*/*v*) was used for staining. The liposomal sample was added to an equal volume of phosphotungstic acid and mixed well [17]. A drop of this sample was placed on the grid and allowed to dry for 2–3 min. Excess liquid was wiped off from the edge of the grid without touching the grid using tissue paper. The sample on the grid was allowed to dry before imaging in a microscope.

Encapsulation efficiency was evaluated by disrupting the liposomes with methanol. Briefly, 1 mL of liposomal sample was added to the volumetric flask (100 mL). The volume was made up to the mark using methanol. The mixture was sonicated for 5 min in a bath sonicator and filtered through a 0.22 μm PVDF filter discarding the first 5 mL of filtrate [18]. The filtrate was analyzed using the RP-HPLC method as detailed subsequently. Six different samples were analyzed from six different batches to check the uniformity and batch to batch variation.

#### 2.2.3. RP-HPLC Analysis of Naringin

The content of naringin was determined using a previously published validated RP-HPLC method [11]. All test samples were diluted suitably with mobile phase, and the chromatographic separation was performed using an isocratic elution. The mobile phase consisted of a mixture of potassium phosphate buffer (10 mM, pH adjusted to 3.6 using dilute orthophosphoric acid) and acetonitrile (25:75) and delivered at a flow rate of 1 mL/min. The HPLC system consisted of a pump (Jasco PU-2080 Plus, Intelligent HPLC pump, Jasco, Tokyo, Japan) connected to Detector (Jasco 2075, Intelligent UV–vis detector, Jasco, Tokyo, Japan). The separation was carried out at 20 °C, on a reversed-phase C18 HPLC column (Qualisil^®^ BDS, 250 mm × 4.6 mm, 5 μm particle size, Qualisil, Agilent Technologies, Mumbai, Maharashtra, India). An injection volume of 20 μL was used. Detections were carried out at 284 nm.

#### 2.2.4. Analysis of Surfactant Functionality by Adsorption Studies

Langmuir−Blodgett (LB) trough instrument (Biolin Scientific, Manchester, UK) was used. The trough was filled with subphase, 10 mM (pH 7.4) phosphate-buffered saline maintained at 37 ± 0.5 °C [6]. Formulation (3 mL) was added and allowed the formation of a uniform monolayer of surfactant molecules. Surface pressure measurements were carried out using a sandblasted gold Wilhelmy plate connected to a microbalance for half an hour (*n* = 3).

#### 2.2.5. Measurement of Airway Patency

Capillary surfactometer (Calmia Medical, Inc., Toronto, ON, Canada) that simulates terminal human airways is used to determine airway patency [14]. A liposomal sample (0.5 μL) was introduced into the narrow section of a glass capillary of internal diameter 0.25 mm. The other end of the capillary was connected to a bellows and a pressure transducer. The liposomal sample was subjected to pass through the capillary under the influence of the steady airflow generated by the continuous compression of the bellows. The changes in the pressure were recorded as % opening time of capillary for 120 s. For comparative analysis, pristine naringin solution and water were also tested similarly. All measurements were carried out in triplicate.

#### 2.2.6. In Vitro Lung Deposition Experiments with the Anderson Twin Stage and the Impinger Cascade Impactor

All glass twin stage impinger (TSI; British Pharmacopoeia Apparatus A, Copley Scientific, Nottingham, UK) paired with a Copley TPK 2000 critical flow controller linked to a Copley HCP5 vacuum pump [19] was used to check the post nebulization droplet size distribution. The upper (stage I) and lower (stage II) chambers of TSI were filled with methanol, 7 and 30 mL, respectively [20]. The liposomal naringin (1 mg/mL, 5 mL) was aerosolized using an Aeroneb^®^ Lab micropump nebulizer fitted at the entrance of TSI [21]. During nebulization, a DFM 2000 flow meter and an HCP5 vacuum pump (Copley Scientific, Nottingham, UK) were utilized to maintain a 60 L/min airflow rate inside the impinger. The procedure was carried out till all the samples added to the nebulization port were nebulized. This procedure took approximately 5.2 ± 0.5 min. After complete nebulization, samples were collected from the neck (area closest to the sample holder), upper stage (stage I), and lower stage (stage II) of the TSI. The content of naringin was determined using the above-mentioned validated HPLC method.

Anderson Cascade Impactor (ACI, Copley Scientific, Nottingham, UK) was used to measure the MMAD. To minimize evaporative loss, all the plates were previously chilled to 10 °C, and then the liposomal formulation was nebulized for 4 min via induction port of ACI using a pump at a flow rate of 15 L/min into the chamber [22]. Samples were taken from each step, including the induction port and filter, by rinsing with methanol and analyzed for naringin content using RP-HPLC, as mentioned earlier. All experiments were carried out in triplicate. MMAD, Geometric standard deviation (GSD), emitted dose (ED), and fine particle fraction (FPF) were calculated by quantifying the liposomal deposition at each stage in the ACI [23,24].

#### 2.2.7. In Vivo Pulmonary Fibrosis Induction in Rats and Treatment Regimen

##### Animals

The study was carried out in male Wistar-albino rats (*n* = 48) with an average body weight of 180–220 g. The rats were housed in standard laboratory conditions (12 h light/dark cycles, 22 ± 2 °C, and 55 ± 5% humidity. Animals were fed with standard pellet chow and water ad libitum. The experiments were conducted at CPCSEA (Committee for the Purpose of Control and Supervision of Experiment on Animals, Bangalore, India) approved animal house. The study protocol was approved by the Vidya Siri College of Pharmacy’s Institutional Animal Ethics Committee for Animal Care and Use (Bangalore, Karnataka, India) with the protocol approval number VSCP/EC/2808/2020/1 and the date of approval 15 February 2021.

##### Induction of Pulmonary Fibrosis in Rats by Bleomycin and Therapy with Liposomal Naringin

The rats (*n* = 12) were randomly divided into four groups, namely (I) Normal control NC, (II) Diseased control (DC, Bleomycin alone), (III) Pristine naringin treatment (NAR, Bleomycin + pristine naringin suspension by inhalation route, 15 mg/kg), and (IV) Liposomal naringin treatment (L-NAR, Bleomycin + liposomal naringin by the inhalation route, 15 mg/kg). After overnight fasting, all the rats were anesthetized with intraperitoneal injections of ketamine–xylazine [25]. Groups II, III, and IV were given a single dosage of 0.1 mL bleomycin (5 mg/kg, 5 mg/mL in 0.9 percent *w/v* normal saline, intratracheal) to induce pulmonary fibrosis. [21]. The process was carried out in the same way for the NC group of normal control rats, with the difference that saline was used instead of bleomycin. Animals from group NAR and L-NAR were treated three times a week with aerosolized pristine naringin suspension and liposomal naringin, respectively, using an Aeroneb^®^ Lab micropump nebulizer (Aeroneb^®^, Kent Scientific Corp., Torrington, CT, USA).

At the end of four weeks, rats were sacrificed, and the thoracic cavity was exposed for collection of bronchoalveolar lavage fluid (BALF). The trachea was exposed and connected to a 16-gauge cannula. Left main bronchi were clamped, and 1 mL sterile chilled normal saline solution at 4 °C was pumped through the cannula into the right lung. It was repeated 2 more times, and the lavage fluid was centrifuged for 10 min at 3500 rpm at 4 °C. The cells precipitated as pellets were collected and re-suspended in 500 μL of sterile saline solution. Left lungs were isolated, rinsed with chilled saline then fixed in 10% buffered formalin for further histopathological investigations. After collection of the BALF, right lungs were immediately preserved at −80 °C and used for determination of hydroxyproline.

#### 2.2.8. BALF Total and Differential Cell Count, Protein Content, and Lactate Dehydrogenase (LDH) Activity

After centrifugation of BALF, the cells pellet was resuspended in 500 μL of normal saline and again centrifuged on slides. The cellular analysis was performed within 1 h of collection of BAL fluid in nutrient-poor media. The total cell count was obtained via a hemocytometer, and cell viability was determined by Trypan blue exclusion. Differential cell counts were performed with Wright-Giemsa staining and enumeration of at least 500 cells. Cell counting was done by observation of a slide in a microscope at a magnification of 100×. Differential cell count was expressed as a number of cells/mL. The total protein content and lactose dehydrogenase levels were determined using commercial kits (Sigma Aldrich, Bangalore, Karnataka, India) by following the manufacturer’s instructions.

#### 2.2.9. Hydroxyproline Quantification in Lung Tissue

Lung tissue was homogenized and centrifuged at 12,000× *g* for 15 min at 4 °C. The supernatant was separated and kept on ice for further use. Hydroxyproline levels in the supernatant were determined using a commercially available kit (Sigma Aldrich, Bangalore, Karnataka, India) following the manufacturer’s protocols and were expressed as μg/g of the right lung.

#### 2.2.10. Study of Antioxidant Biomarkers and Oxidative Stress in Lung Tissues

A commercially available kit was used to measure superoxide dismutase (SOD) (Cayman Chemical Company, Ann Arbor, MI, USA). The lung tissue homogenate was tested by following the manufacturer’s instructions. SOD activity was measured in U/mg protein. This kit detects superoxide radicals produced by xanthine oxidase and hypoxanthine using a tetrazolium salt. Glutathione peroxidase (GPx) activity from lung tissue homogenate was measured by using a commercially available kit (Cayman Chemical, Ann Arbor, MI, USA). The level of GPx activity was measured in tissue and represented as nmol/min/mg protein [9].

#### 2.2.11. Histopathological Analysis of Lung Tissues Stained with Hematoxylin and Eosin (H and E) and Masson’s Trichrome Stains and Other Vital Organs Stained with H and E

The lungs of 6 randomly selected rats were preserved in 10% formalin in phosphate-buffered saline for 24 h and sectioned (4 μm) after inserting in a paraffin block. To determine the extent of lung tissue destruction, the slices were stained with H and E and Masson’s Trichrome [26]. The slices were examined under a light microscope and analyzed in random order by a pathologist who was blinded to the experimental circumstances. The severity of emphysema, the thickness of the alveolar wall, fibrosis, and collagen deposition or inflammatory lesions were all evaluated histologically in lung tissue. Depending on the level of alveolitis and fibrosis, the location and severity of these lesions were assessed. The scale ranged from 0 to 3, score 0 indicative of the absence of alveolitis/fibrosis, whereas mild, moderate, and severe distribution were scored as 1, 2, and 3, respectively [27].

#### 2.2.12. Statistical Analysis

In all experiments, the results are presented as mean standard deviation. The statistical significance of the findings was determined using a one-way analysis of variance (ANOVA) with a 95% confidence level. For assessing any significant differences between groups Newman–Keuls test for statistical significance at 95% confidence limit was used.

## 3. Results and Discussion

In the current battle of the COVID-19 pandemic, where the world has witnessed a huge loss of life, the challenging part is to manage the sequelae like lung fibrosis. Elderly patients, critically ill patients, and those on mechanical ventilation are at the highest risk of developing lung fibrosis [28]. The complex etiology of pulmonary fibrosis and the nonavailability of effective therapy make management of pulmonary fibrosis an unmet medical need. Poor prognosis, limited treatment options available, and the progressive nature of the disease leading to a rapid decline in lung function results in a high fatality in pulmonary fibrosis [29].

Although the origin of fibrosis remains elusive, an imbalanced alveolar redox status in the lung with increased oxidative stress, increased flux of inflammatory cytokines and enzymes, and decreased levels of endogenous antioxidants are the common observations of the fibrotic lung [30]. It results in continuous ongoing injury to the alveolar epithelium that causes surfactant system dysfunction [31] and decreases the regeneration capacity, finally resulting in alveolar collapse and fibroproliferative consequences [29]. Additionally, chronic surfactant dysfunction leads to abnormally high surface tension that further introduces mechanical stress, thickening of the septal wall, and collagen deposition in the lungs [32]. Therefore, biophysically active surfactant-based local delivery of drug would help to reduce the surface tension associated with mechanical stress at the alveolar interface and also prevent alveolar collapse [29,33].

Growing in vivo and in vitro data indicates that naringin’s anti-inflammatory action may help reduce oxidative stress-related illnesses, including pulmonary fibrosis. [10,34]. However, there have been no studies on the use of naringin in combination with a pulmonary surfactant for the treatment of pulmonary fibrosis, probably due to its poor aqueous solubility that limits in vivo bioavailability. Lipid-based delivery systems like liposomes enhance the performance of the incorporated bioactive agents by improving their solubility and bioavailability, in vitro and in vivo stability, and also prevent unwanted interactions with other molecules. Another advantage of liposomes is their biocompatibility and ability to scale up for commercial production [35]. For pulmonary delivery, liposomes made from exogenous surfactants are best as they are rapidly adsorbed at the air-liquid interface in the lungs that accentuates the ability of the liposomes to open up, forming a monolayer film, and spread at the interface [36]. This facilitates in delivery of encapsulated drugs to the alveolar interface and especially to the collapsed alveoli due to the surfactant action [14]. Our study systematically explored the feasibility of aerosolized delivery of liposomal nano-carrier of naringin and provided the proof-of-concept for assist in pulmonary mechanics and improved therapeutic efficacy.

### 3.1. Physicochemical Characterization

**Figure 1:**
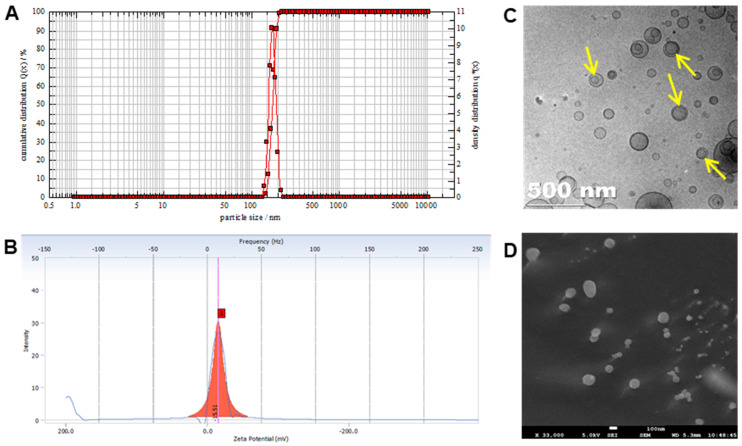
Characterization of liposomal naringin. (**A**) Particle size analysis, (**B**) Zeta potential, (**C**) TEM image with arrows indicating the lamellae of vesicles, and (**D**) SEM image.

The mean particle size and PDI of liposomal naringin were 171.4 ± 5.8 nm and 0.2 ± 0.012, respectively (Figure 1A). Biodegradable and biocompatible liposomes for pulmonary delivery offer the benefit of entrapment of lipophilic therapeutic molecules in vesicles which on inhalation help in localizing the drug effect in the pulmonary system for a longer duration of time. This enhances the therapeutic benefit while reducing the possibility of systemic adverse effects [36]. In the context of pulmonary drug delivery, safety and controlled release are ideal for liposomes incorporating anti-fibrotic agents because phospholipid carriers have no toxic effects, and the action is aimed to be confined to the lungs [37]. The negative zeta potential of −15.5 ± 1.3 mV (Figure 1B) indicates electrostatic repulsion owing to a significantly negative surface charge responsible for the stability of the liposomes. TEM and SEM (Figure 1C,D) imaging revealed that the liposomal particles are less than 200 nm diametrically with uniform spherical non-agglomerating structures made up of unilamellar phospholipid bilayer.

High-pressure methods are considered more efficient processes for the large-scale production and size reduction of lipid vesicles that could overcome the limitations of traditional production methods of liposomes. Liposomes of nanometric dimensions can be easily obtained with better control of the particle size distributions and more than 90% encapsulation efficiencies [38]. In the present study, encapsulation efficiency was found to be 91.5 ± 2.4%. The high entrapment efficiency of lipophilic naringin is attributed to the small unilamellar structure of liposomes and the high lipid-to-drug ratio.

### 3.2. Surfactant Functionality

The pulmonary surfactant system is comprised of lipids that contribute to pulmonary mechanics and stabilize alveoli even at lower lung capacities [39]. Pulmonary fibrosis is linked with surfactant system disfunction, the collapse of distal airspaces as well as collapse induration which leads to altered lung function [31,40]. The surfactant property of liposomal lipids highlights the liposomes’ ability to open up to create a monolayer film and distributes at the interface like surfactant vesicles in vivo. The formulation’s rapid adsorption at the air-fluid interface indicates the appropriate surfactant quality needed for effective pulmonary administration [12] and is important to maintain airway patency. This property will aid in the delivery of naringin to the interface in the deep alveoli and will also assist in the delivery of the drug to the collapsed alveoli.

**Figure 2:**
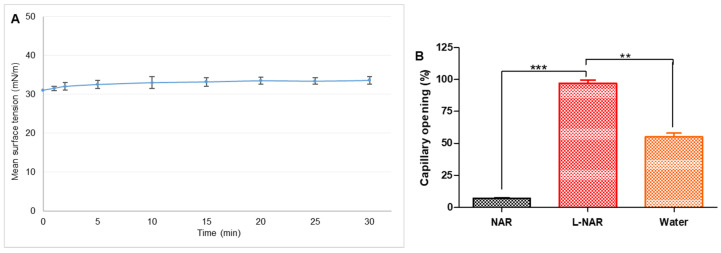
(**A**) Surface tension-time isotherms indicating adsorption of liposomal naringin. Each value represents the mean of 3 determinations. (**B**) Capillary patency of pristine naringin (NAR), liposomal naringin (L-NAR), and water measured using capillary surfactometer. The data is presented as the average of three determinations. Error bars represent standard deviation. **, and *** show significant difference by Newman–Keuls analysis following ANOVA at 95 percent confidence level at *p* < 0.01 and *p* < 0.001, respectively.

Inhalation of aerosols that interfere with surface activity at the air-fluid interface would increase surface tension. It will eventually produce pulmonary edema by increasing the vascular permeability [41]. The contents of edematous fluid further destroy the surfactant present at the air-fluid interface in the lungs. Therefore, it is highly desirable to have surfactant-like activity in aerosol-based formulations. LB instrument was used to evaluate the surface tension of the liposomal naringin formulation. Surface tension achieved by the formulation as a lipid structure surface explains the tendency of the formulation to adsorb over the air−water surface. The measured surface tension of the liposomal naringin was 32.6 ± 0.96 mN/m (Figure 2A).

Naringin liposomes exhibited efficient surfactant function apart from acting as a pulmonary drug delivery system. Smaller size and higher surface area/volume ratio in cases of nanosized liposomes can increase the number of liposomes reaching the interface and getting adsorbed, which would further enhance the rate of surface tension reduction.

### 3.3. Airway Patency

Pulmonary surfactant, an amphiphilic lipoprotein complex, lowers the surface tension in the alveoli of the lungs, maintains terminal conducting airways patency, provides low airway resistance, and promotes blood-gas exchange. In clinical conditions like pulmonary fibrosis, lack of surfactant protein complex results in the replacement of normal lung tissue with excess connective tissue and limiting oxygen diffusion across the alveolar-capillary membranes [42]. Therefore, an inhalation-based drug delivery system needs to possess surfactant activity to facilitate airway patency and ensure the spread of the formulation to terminal airways. With a well-functioning surfactant, the capillary opens up without offering any resistance to air, and thus pressure exerted is recorded as zero and capillary opening will be 100%. The naringin liposome formulation was instilled in the narrow-constricted portion of the capillary (0.25 mm internal diameter) that mimics the terminal airways. Air was forced through the occluded capillary that caused the expulsion of the sample from the constricted portion. If the naringin liposomes exhibited strong surface activity, as the formulation did not return to the small capillary section and maintained airway patency of 97 ± 2.5% for 120 s, it was similar to natural lung surfactants (L-NAR, Figure 2B). Pristine drug solution offered resistance and could not maintain capillary patency as observed (7.1 ± 0.5%, NAR, Figure 2B). In an already compromised respiratory condition like pulmonary fibrosis, the ability to maintain airway patency could prove vital in providing symptomatic relief.

### 3.4. In Vitro Lung Deposition

**Figure 3:**
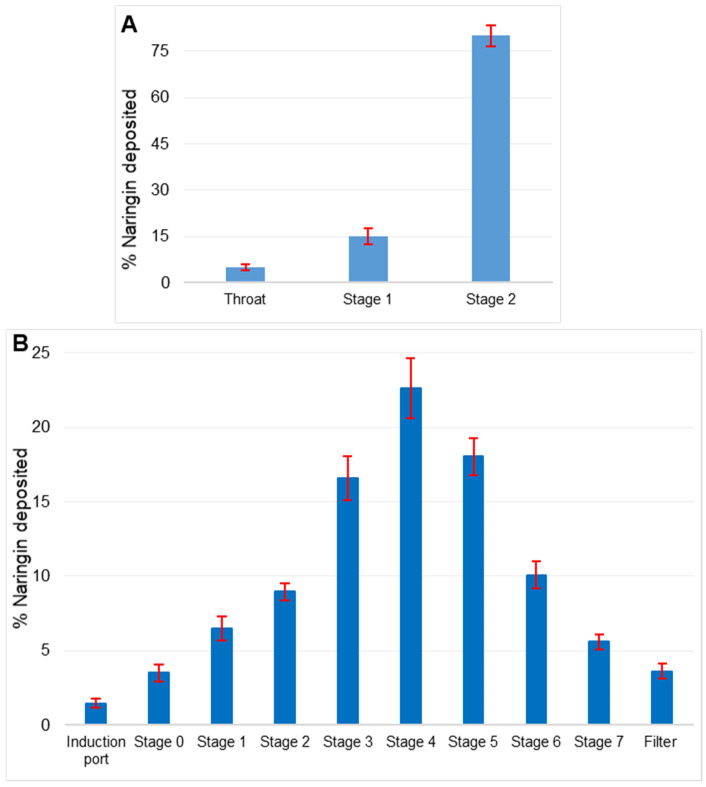
(**A**) In vitro lung deposition using Twin Stage Impinger (TSI) depicting deposition in different parts. (**B**) Anderson Cascade Impactor (ACI) deposition pattern of liposomal naringin on various stages of impactor at a flow rate of 15 L/min. Data is represented as a mean of 3 determinations. Error bars indicate standard deviation.

The TSI is a simple device that mimics the behavior of pulmonary aerosol particles in a deflected airstream colliding with liquid surfaces due to their inertia. TSI has shown to be useful in the fast screening of aerosol deposition patterns. [43]. Stage 2 of the twin stage impinger demonstrated deposition of 79 ± 1.5% of the formulation, suggesting reduced airway delivery. Only 5% of the nebulized aerosol was deposited on the throat (Figure 3A).

MMAD and GSD of the nebulized naringin liposomal nanosuspension were measured by ACI (Figure 3B) and were 2.35 ± 1.02 μm and 1.26 ± 0.80 μm, respectively. The ED and FPF of the nebulized product were 94.88 ± 0.51% and 85.10 ± 1.03%, respectively. The greater FPF, smaller MMAD, and GSD imply stronger lung deposition, optimal aerosol particle size, and narrower particle size dispersion of the aerosol droplets. MMAD of 1−5 μm is considered ideal for delivery to deep lungs with minimum oropharynx deposition [44]. The results suggest that liposomal naringin possesses good aerosolization properties and is suitable for deep lung tissue drug delivery.

### 3.5. In Vivo Pharmacodynamic Efficacy of Liposomal Naringin in Bleomycin-Induced Lung Fibrosis Model in Rats

#### 3.5.1. Total and Differential Cell Count, Protein Content and LDH

**Figure 4:**
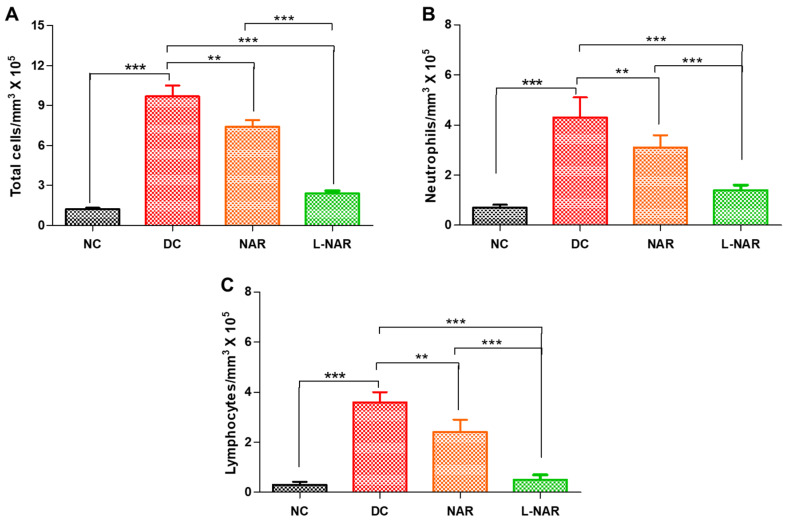
Effect on inflammatory cell counts in the BALF of rats, normal control (NC), disease control (DC), inhalation therapy of pristine naringin (NAR), and liposomal naringin (L-NAR) on with pulmonary fibrosis induced using intratracheal bleomycin. Total cells (**A**), Neutrophils (**B**) and Lymphocytes (**C**). The results were given as the mean standard error of the mean (*n* = 12). **, and *** show significant difference by Newman–Keuls analysis following ANOVA at 95 percent confidence level at *p* < 0.01 and *p* < 0.001 respectively.

In pulmonary fibrosis, the activation of macrophages and massive influx of neutrophils cause lung alveolitis and epithelial cells deterioration [21]. These cells affect alveolar/capillary barrier permeability that causes edema. The intratracheal instillation of bleomycin significantly elevated the total cell count (Figure 4A), neutrophils (Figure 4B), and lymphocytes (Figure 4C) in BALF compared to normal control (*p* < 0.001). Inhalation therapy with naringin thrice a week significantly reduced total cell count, neutrophils as well as lymphocytes compared to the diseased control (DC) group. Pristine naringin could show some effect in reducing the inflammatory cell count, liposomal naringin was significantly more effective (*p* < 0.001).

**Figure 5:**
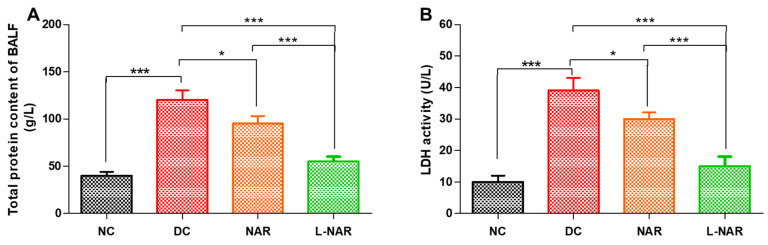
Total protein (**A**) and Lactate dehydrogenase (LDH) activity (**B**) in BALF, from various groups, namely normal control (NC), disease control (DC), inhalation therapy of pristine naringin (NAR), and liposomal naringin (L-NAR) (*n* = 12). *, and *** indicate significant difference by Newman–Keuls analysis after ANOVA at 95 percent confidence level at *p* < 0.05, and *p* < 0.001 respectively.

Protein leakage is another indicator of the inflammation induced by bleomycin. Cytoplasmic cellular enzyme LDH released from damaged inflammatory cells into the extracellular space specifies the disturbances of the cellular integrity induced by inflammatory pathological condition and pulmonary endothelial lung injury [45]. It is also a marker of hypoxia and interstitial lung deterioration. Bleomycin instillation in rats resulted in increased protein concentration (Figure 5A) and LDH activity (Figure 5B) in BALF. Naringin displayed its anti-inflammatory effect and reduced both LDH activity and total protein content.

#### 3.5.2. Hydroxyproline Content and Levels of Antioxidant Enzymes SOD and GPx

**Figure 6:**
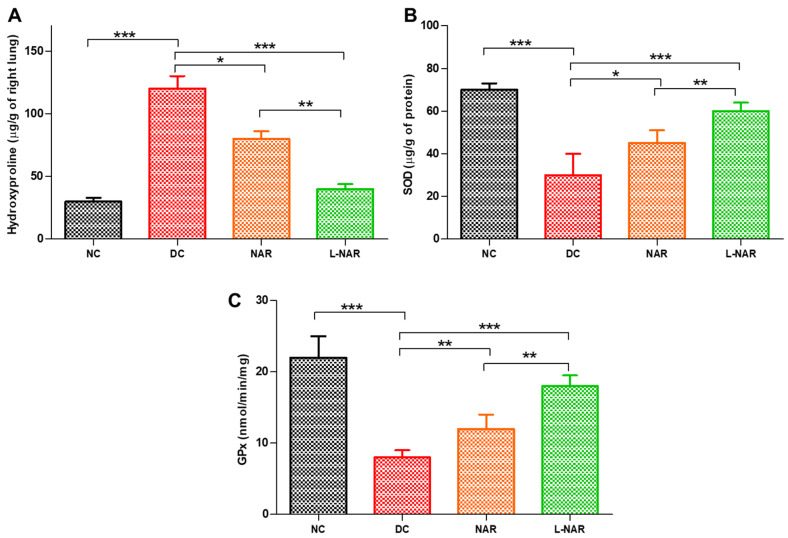
Hydroxyproline (**A**), Superoxide dismutase (SOD, (**B**)), and Glutathione peroxidase (GPx, (**C**)) activity from various groups only normal control (NC), disease control (DC), inhalation therapy of pristine naringin (NAR), and liposomal naringin (L-NAR) (*n* = 12). *, ** and *** indicate significant difference by Newman–Keuls analysis after ANOVA at 95 percent confidence level at *p* < 0.05, *p* < 0.01 and *p* < 0.001 respectively.

During the progression of lung fibrosis, phenomenological changes occur in the extracellular matrix. Excessive deposition of hydroxyproline, a non-proteinogenic amino acid, is synthesized by the post-translational hydroxylation of proline during collagen biosynthesis serves as a biochemical marker of fibrosis [46]. Bleomycin is a potent inducer of free radicals like superoxide and hydroxyl radicals and also stimulates the synthesis of collagen. Cytokine dysregulation causes activation of fibroblasts and inflammation that inhibit collagen degradation leading to a rise in collagen levels. Bleomycin significantly increased collagen levels (DC, Figure 6A).

Treatment with liposomal naringin decreased it significantly, indicating a beneficial effect of formulation in disease recovery. (L-NAR, Figure 6A). This effect can be related to the antioxidant and anti-inflammatory effect of naringin and the ability of the formulation to reach deep alveoli due to its surfactant effect. Naringin can restore the levels of antioxidant enzymes by scavenging free radicles and thus reduces oxidative stress [47]. SOD and GPx are the body’s important antioxidant enzymes that help reduce reactive oxygen species and related damage. GPx is a selenium-dependent and independent antioxidant enzyme that protect the lungs from damage caused by reactive oxygen species and prevent the formation of peroxides. These enzymes neutralize damaging hydrogen peroxide as well as a large variety of hydroperoxides into safe by-products and thus protect tissues against oxidative stress. Liposomal naringin effectively restored the levels of SOD and GPx in fibrotic lungs (Figure 6B,C).

#### 3.5.3. Tissue Histopathology

**Figure 7:**
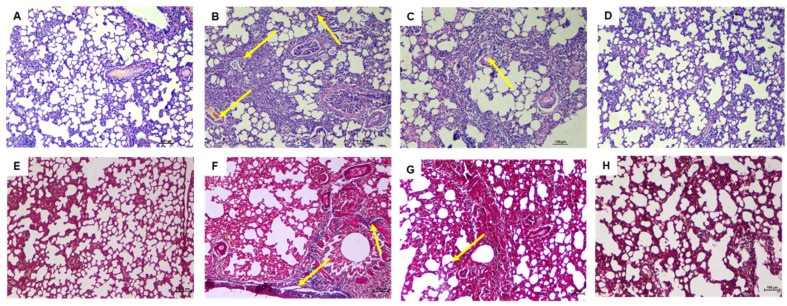
Effect of daily inhaling therapy of liposomal naringin evaluated utilizing lung histological changes using H and E staining (100×). (**A**) Normal control, the lung architecture is intact, with no indications of injury, (**B**) Disease control, showing moderate fibrotic changes, (**C**) Pristine naringin therapy revealing mild to moderate fibrotic changes, and (**D**) Liposomal naringin treatment with the restoration of normal lung architecture. Masson’s Trichrome staining was used to examine lung histological changes (100×). (**E**) The lack of blue staining in the normal control indicates the absence of collagen deposition. (**F**) Disease control demonstrates the blue color of collagen deposition. (**G**) Pristine naringin treatment revealed mild bluish coloration of collagen, and (**H**) Liposomal naringin treatment showed significantly lesser deposition of collagen.

As seen under the microscope, the H and E stained lung tissue samples of the normal group (Figure 7A) showed normal tissue and cell structure, while the diseased control group (Figure 7B) that received bleomycin alone showed moderate hemorrhagic changes, fibroplasia, and regions of increased alveolar septal thickness, emphysema, and leukocytic infiltration in the alveolar walls.

Treatment with pristine naringin (Figure 7C) for 1 month showed mild to moderate degree of septal thickening with infiltration of a few inflammatory cells, emphysematous changes, and alveolar hemorrhage. Treatment with liposomal naringin (Figure 7D) via inhalation route helped the restoration of normal lung architecture with very few signs of inflammation, edema, bleeding, emphysema, or fibrosis. Masson’s trichrome staining of lungs of normal control group rats revealed the absence of blue staining denoting no collagen deposition in interstitial spaces as well as alveolar septae (Figure 7E), (Figure 7B) Disease control showing bluish coloration of collagen deposition at interstitial space and peri-bronchial area, (Figure 7C) Pristine naringin treatment revealing mild bluish coloration of collagen deposition at interstitial space and peri-bronchial area, and (Figure 7D) Liposomal naringin treatment showing significantly lesser deposition of collagen. Histopathology of other vital organs, namely liver, kidney, brain, heart, spleen, adrenal, and thymus gland, showed no tissue abnormalities indicating that naringin treatment is not only effective for pulmonary fibrosis but is also safe for other organs (Supplementary Figure S1).

## 4. Conclusions

The development of successful nanotechnology-based products for pulmonary delivery is always a challenge. Particles with MMAD < 5 µm are required to reach the mid and deep lung parenchyma. At the same time, particles with a size less than 500 nm are easily exhaled. Therefore, for a successful nanoscales delivery system, several parameters play an important role like particle size, formulation composition, particle fluidity, surface chemistry, etc. [41].

Although nintedanib and pirfenidone changed the treatment paradigm in pulmonary fibrosis and increased our understanding of mechanisms of disease progression, several challenges and unmet needs still need to be managed to improve the quality of life of patients. Growing evidence in the literature indicates surfactant dysfunction, inflammation, and the influx of a plethora of inflammatory cytokines and enzymes in fibrotic tissue of lungs during the progression of the disease [9]. However, to date, there is no report on the development of a product that could act in a multifactorial way for the management of pulmonary fibrosis. We present a holistic approach of the use of pulmonary surfactants to develop nanosized liposomes encapsulating antioxidant phytochemicals, naringin that exhibits multi-pathway interference in the process of inflammation. Considering the chronic disease, we opted for a non-invasive and patient-friendly aerosol system that would help to improve compliance to therapy. The aerosol inhalation of the liposomal naringin achieved optimal formulation parameters like high entrapment efficiency, size, zeta potential airway patency, and in vitro lung deposition. Therapeutic results in the bleomycin-induced lung fibrosis animal model suggested the effectiveness of the liposomal naringin by the inhalation route. Therefore, the easy-to-scale up formulation of liposomal naringin using pulmonary surfactants offers a potential platform for aerosol delivery, thereby expanding the clinical application in the treatment of pulmonary fibrosis in the future. The present formulation presents a versatile technology that can be optimized to incorporate a variety of hydrophobic drugs that will allow effective localized delivery of the therapeutic agents in the management of various lungs diseases like acute respiratory distress syndrome, acute lung injury, lung cancer, and chronic obstructive pulmonary disease.

## Data Availability

All the relevant data is included in the manuscript.

## Supplementary Materials

The following are available online at https://www.mdpi.com/article/10.3390/pharmaceutics13111851/s1, Figure S1: Representative histopathology images of vital organs of the treated group number IV.
