# Supplementary material for: Aerosol Delivery of Surfactant Liposomes for Management of Pulmonary Fibrosis: An Approach Supporting Pulmonary Mechanics
Source: Pharmaceutics. 2021 Nov 3;13(11):1851. doi: 10.3390/pharmaceutics13111851 (PMC8625129; doi:10.3390/pharmaceutics13111851)
Supplement: Supplementary file 1 [file pharmaceutics-13-01851-s001.zip › pharmaceutics-1431027-supp-revised.pdf]

pharmaceutics

MDPI

Supplementary Materials: Aerosol Delivery of Surfactant Liposomes for Management of Pulmonary Fibrosis: An Approach Supporting Pulmonary Mechanics

Sabna Kotta, Hibah Mubarak Aldawsari, Shaimaa M. Badr-Eldin, Lenah S Binmahfouz, Rana Bakur Bakhaidar, Nagaraja Sreeharsha, Anroop B. Nair and Chandramouli Ramnarayanan

Organ	Representative Histopathology Image H and E Stained
Liver	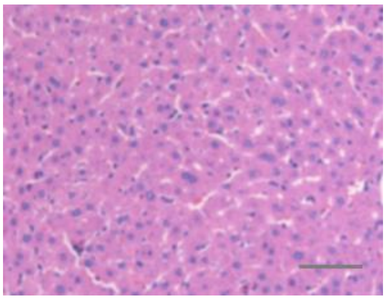
Kidney	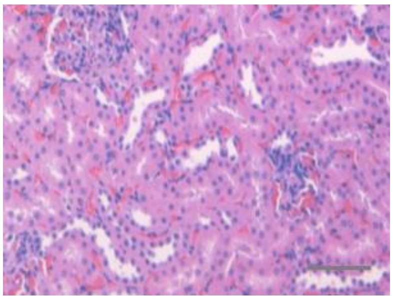
Spleen	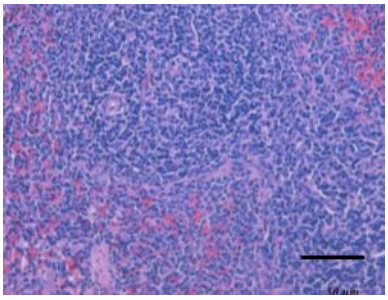
Adrenal gland	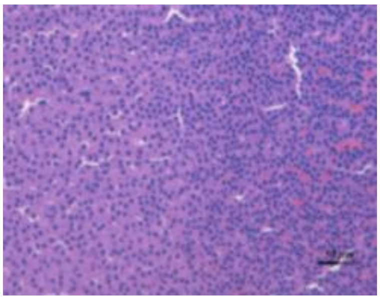

**Brain**	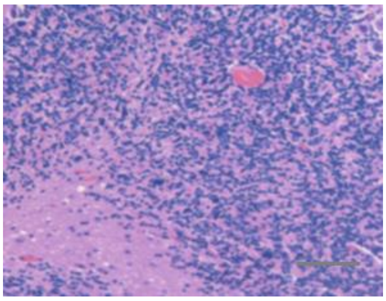
**Heart**	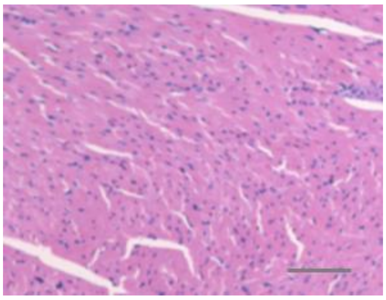
**Thymus**	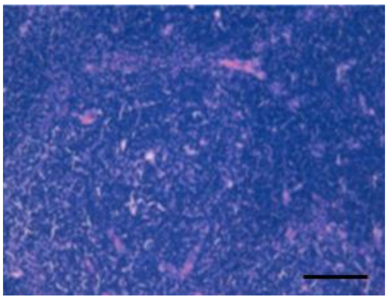

Figure S1. Representative histopathology images of vital organs of the treated group number IV. (Scale bar 0.5 mm = 50 µm)
